# Assessment of sensorimotor and functional differences between patients with surgically treated partially incongruent Lisfranc injuries and healthy controls: a cross-sectional study

**DOI:** 10.1007/s00402-025-05947-0

**Published:** 2025-06-04

**Authors:** Ali Ilez, Abdullah Kahraman, Muhammet Ibrahim Karaçam, Türker Şahinkaya, Mehmet Demirel, Gökhan Polat, Defne Kaya Utlu

**Affiliations:** 1 Faculty of Health Sciences, Department of Physiotherapy and Rehabilitation, Istanbul Beykent University, Istanbul, Turkey; 2 Istanbul Faculty of Medicine, Department of Orthopaedics and Traumatology, Istanbul University, Istanbul, Turkey; 3 Istanbul Faculty of Medicine, Department of Sports Medicine, Istanbul University, İstanbul, Türkiye; 4 Faculty of Health Sciences, Department of Physiotherapy and Rehabilitation, University of Health Sciences, İstanbul, Turkey

**Keywords:** Lisfranc injuries, Plantar sensation, Ankle proprioception, Mobility, Balance, Isokinetic strength

## Abstract

**Introduction:**

Lisfranc injuries are relatively rare but associated with a notoriously poor prognosis despite significant advances in their surgical treatment. This study aimed to investigate changes in plantar sensation, ankle proprioception, mobility, balance, and isokinetic strength in patients treated by open reduction and internal fixation (ORIF) for partially incongruent Lisfranc injuries.

**Materials and methods:**

This retrospective cross-sectional study was conducted at a tertiary care hospital. The patient group consisted of 12 patients (9 males, mean age = 39.16 ± 13.57 years) who were treated with ORIF for a Myerson type B partially incongruent Lisfranc injury between 2020 and 2023. Measurements were conducted at an average of 20 (20–42) months post-surgery. The control group included 11 healthy participants (8 males, mean age: 37 ± 8.69 years) with no known orthopedic conditions. Sole sensation, ankle proprioception, ankle dorsiflexion (DF) and plantarflexion (PF) muscle strength and endurance were measured with CYBEX 350 isokinetic dynamometer, along with dynamic balance, ankle mobility, and PF endurance.

**Results:**

The patient group exhibited significant reductions in mid-foot plantar sensation (*p* = 0.019), as well as impairments in active angle replication at 7° DF, 7° PF, and 14° PF (*p* = 0.049, *p* < 0.001, *p* < 0.001, respectively). Additionally, marked declines were observed in both dorsiflexion strength and endurance (*p* < 0.001) and plantarflexion endurance (*p* = 0.007). Furthermore, this group demonstrated decreased dynamic balance (*p* = 0.021), particularly in the anterior direction (*p* = 0.001), alongside diminished ankle mobility (*p* = 0.001) and isotonic endurance of the ankle plantar flexor muscles (*p* = 0.001).

**Conclusions:**

Ankle mobility, balance, and muscle endurance can be limited as a result of substantial proprioceptive losses, decreased midfoot plantar sensation, and poor active angle replication following surgery for Lisfranc injury, according to this study. Emphasizing focused proprioception and balance training in post-operative rehabilitation is crucial to enhance recovery and prevent long-term complications.

## Introduction

Lisfranc injuries encompass damage to the bones or ligaments within the tarsometatarsal and intercuneiform joint complex, ranging from a stable, mild sprain to a significantly displaced and unstable fracture or fracture-dislocation of the midfoot [1]. While stable injuries can be treated non-operatively, surgical treatment is indicated for unstable (displaced) Lisfranc injuries [2]. Open reduction and internal fixation (ORIF) with transarticular screw fixation has been considered the gold-standard surgical method [2]. Lisfranc injuries are rare, but they are frequently associated with significant post-operative morbidity, even after optimal surgical treatment. Common post-operative complications include post-traumatic arthritis, joint stiffness, persistent pain, and impaired gait mechanics due to altered plantar pressure distribution [2–4]. Additionally, proprioceptive deficits and dynamic balance impairments may persist, affecting overall functional recovery [5, 6]. Given these challenges, long-term rehabilitation strategies focusing on proprioception and functional mobility are crucial for improving patient outcomes.

Despite these known complications, most studies evaluating Lisfranc injuries have focused on radiographic outcomes, plantar pressure changes, and general functional scores [4–11]. However, there is a lack of research specifically assessing proprioceptive impairments and dynamic balance deficits, which are critical for post-injury mobility and overall recovery. Addressing this gap is essential, as proprioceptive dysfunction and balance impairments may increase the risk of secondary injuries and long-term disability. In addition to radiographic and functional assessments, evaluating protective sole sensation, ankle proprioception, and muscle strength is crucial for understanding the long-term functional consequences of Lisfranc injuries. Protective foot sensation plays a key role in balance and postural control, and its impairment may contribute to gait instability [12]. Ankle proprioception is essential for coordinated movement and injury prevention [13, 14], yet its alterations following Lisfranc injuries remain largely unexplored. Muscle strength deficits, particularly in the foot and lower limb, can further exacerbate mobility impairments and functional limitations [8]. Despite their clinical significance, these aspects have not been thoroughly investigated in patients treated with ORIF for Lisfranc injuries, highlighting the need for a comprehensive assessment.

The aim of this study was to investigate and compare the protective sole sensation, ankle proprioception, muscle strength and endurance, and dynamic balance of patients treated with ORIF after partially incongruent Lisfranc injuries against healthy groups.

## Materials & methods

### Study population

This retrospective study included twelve patients treated with ORIF for Type B partial incongruous Lisfranc injuries (as per the Myerson classification) in a single tertiary center between 2020 and 2023. According to Myerson’s classification, Type B Lisfranc injuries are partially incongruous tarsometatarsal (TMT) joint dislocations, where there is a partial displacement of the metatarsals in either the medial or lateral direction without complete disruption of the Lisfranc joint complex. These injuries are further divided into: B1 (Medial Partial Incongruity): Displacement of the first metatarsal and medial column (medial cuneiform ± second metatarsal), with the medial column shifting away from the middle and lateral columns. Inter-cuneiform instability may be present. B2 (Lateral Partial Incongruity): Lateral displacement of the second to fifth metatarsals, disrupting alignment with the cuboid while the medial column remains stable. May involve rotational or sagittal plane deformities [11].

All measurements were performed on both lower limbs of all participants by a single experienced physical therapist to ensure consistency. The study was approved by the clinical research ethics committee. Written informed consent was obtained from all participants. The inclusion criteria for the patient group included a minimum of 20 months post-operative follow-up, a diagnosis of Type B partial incongruous Lisfranc injuries, undergoing ORIF for the treatment, and willingness to participate in the study [7, 8]. All functional assessments and questionnaires were conducted on patients who had completed at least 20 months of postoperative follow-up, with the follow-up duration ranging from 20 to 42 months. None of the patients underwent a structured post-operative rehabilitation program, and no specific interventions were implemented to prevent joint adhesions. The exclusion criteria included a history of surgery on the contralateral lower extremity, neurological injury, fractures, or peripheral neuropathy due to conditions such as diabetes. This study included two groups for comparative analysis: Group I (patients with Lisfranc injuries) and Group II (healthy controls). Group I consisted of twelve patients who met the inclusion criteria outlined above. Group II was composed of healthy volunteers with no known orthopedic conditions, meeting the same exclusion criteria.

### Protective foot sensation measurement

**Fig. 1:**
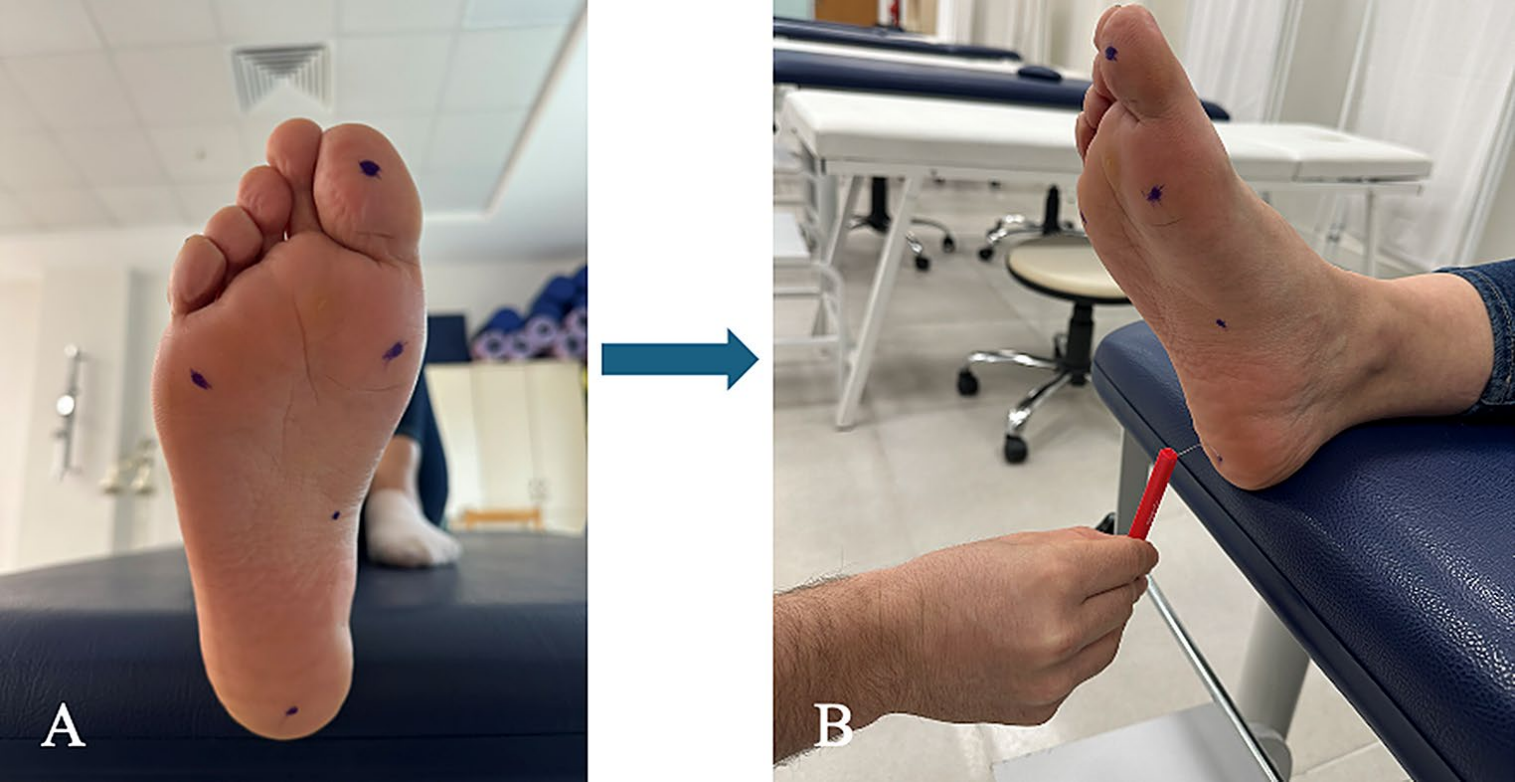
Foot sensation assessment. (**A**) Marked assessment points on the plantar surface of the foot. (**B**) Sensory evaluation using a monofilament test to assess protective sole sensation

The Semmes-Weinstein Monofilament (SWM) test kit (North Coast Medical, San Jose, CA, USA), with monofilaments ranging in size from 1.65 to 5.07, was used to measure protective sole sensation. Five measurement points were selected on the sole: the hallux, the first metatarsal head, the fifth metatarsal head, the midfoot, and the heel [15]. At each measurement point, the relevant area was touched randomly for 1.0–1.5 s, with each point being contacted three times until the monofilament bent into a C-shape. Data were recorded as the monofilament value for that point when the participant correctly responded to all three contacts [15, 16] (Fig. 1).

### Ankle proprioception measurement

**Fig. 2:**
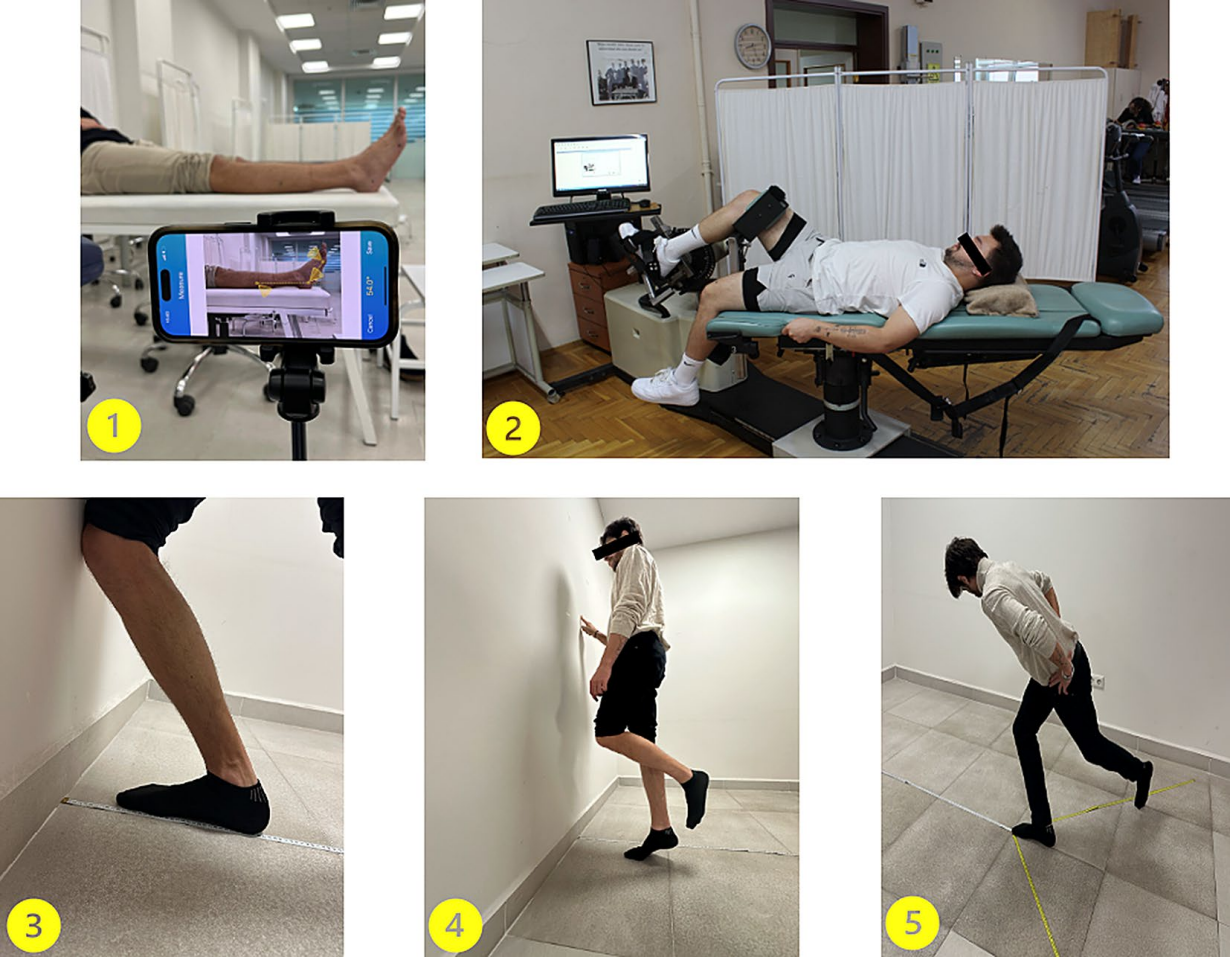
Illustrations of clinical assessment protocols. ^1^Ankle proprioception assessment using active joint repositioning tasks recorded with a digital goniometer and smartphone application. ^2^ Isokinetic strength testing of plantarflexor and dorsiflexor muscles at 30°/s and 120°/s angular velocities using an isokinetic dynamometer.^3^ Weightbearing Lunge Test (WBLT) for evaluating ankle dorsiflexion mobility.^4^Heel-Rise Test (HRT) to assess calf muscle endurance and strength.^5^ Modified Star Excursion Balance Test (MSEBT) for dynamic postural control and balance performance evaluation

An active angle reproduction (AAR) test was conducted to evaluate proprioception in both the DF and PF directions of the ankle. The target angles employed were 7^0^ degrees for DF and 7^0^, 14^0^, and 21^0^ degrees for PF [17]. Participants were positioned in a supine position and instructed to close their eyes during the test to eliminate visual input. The target angles were then actively demonstrated to the participants [18]. The physical therapist stood laterally to the participant. Markers were placed on the fibular head, lateral malleolus, and above the fifth toe while a smartphone recorded the session from a fixed location. The participant moved to a predetermined angle and held it for 10 s. After returning to neutral, the participant repeated the angle recorded by the DrGoniometer app. (CDM s.r.l., Milan, Italy) [19, 20] (Fig. 2). The absolute error score was calculated as the difference between the target and replicated angles with six measurements per angle averaged for the final score [21].

### Muscle strength measurement

Muscle strength of PF and DF were measurement using a CYBEX 350 isokinetic dynamometer (CYBEX HUMAC, version 2009; Computer Sports Medicine Inc., Stoughton, MA). The measured limb was elevated and stabilized just above the knee, behind the thigh (Fig. 2). Measurements were taken on both sides. Prior to testing, participants completed a 10-minute warm-up on a bicycle ergometer (Ergomedic 818E, Monark, Sweden) at 50 W (60RPM). Participants were then instructed to perform four repetitions of ankle DF and PF at maximum effort at angular velocities of 30 and 120 degrees per second [22]. An angular velocity of 30 degrees/s represents maximal voluntary contraction and maximal power, commonly cited in the literature [23, 24]. A velocity of 120 degrees/s was chosen, as high angular velocities are required during daily activities and the normal gait cycle [25].

### Balance measurement

The Modified Star Excursion Balance Test was used to assess dynamic balance. All the study participants watched a video demonstration before starting the test. The angles between the tape measures were set at 135° between anterior-posterior and anterior-lateral and 90° between posterior-lateral [26, 27]. Participants placed their big toe at the center point with their hands on their hips and extended the free limb in the specified direction (Fig. 2). The contact point on the tape measure was recorded in centimeters. After three repetitions, the average of the last three repetitions was recorded. Each direction was assessed sequentially for each leg to prevent fatigue. If the participant raised their hands from their hips, moved the fixed leg, or lost balance, the test was stopped and repeated [27].

### Ankle mobility measurement

The weight-bearing lunge test was used to assess ankle mobility according to the knee-to-wall principle [28]. Participants were instructed to stand with the tested foot facing the wall, with a tape measure placed on the floor. The controlateral foot was positioned one step behind the tested foot, and the hands were placed on the wall [29] (Fig. 2). Participants then attempted to touch the wall with their knee while maintaining heel contact with the floor. The distance between the tested foot and the wall was increased in 1 cm increments until the heel and knee contact could no longer be maintained [29]. The maximum distance, measured in centimeters, was defined as the distance from the big toe to the wall when the knee maintained contact, and the heel did not lift [30].

### Evaluation of functional performance and patient-reported outcomes

The functional level of the participants was determined using the physical performance (heel-rise) test and patient-reported outcome scores American Orthopaedic Foot and Ankle Society (AOFAS) Midfoot Rating System and Foot and Ankle Outcome Score (FAOS)).

AOFAS was utilized, where respondents were rated their pain, function, and alignment on a scale of 1 to 10, with 10 representing the best possible outcome. The AOFAS score is categorized out of 100 points as “excellent” (90–100), “good” (80–89), “fair” (60–79), and “unsatisfactory” (less than 60) [31]. The FAOS is a patient-reported outcome measure that evaluates symptoms, stiffness, pain, functional abilities in daily activities, work-related functions, sports participation, and overall quality of life. It consists of five distinct subscales: Pain, Symptoms, Activities of Daily Living (ADL), Sport/Recreation, and Foot and Ankle–Related Quality of Life (QoL). Each subscale score was obtained by summing the raw item scores within the respective domain and transforming the total into a 0–100 scale, where 100 indicates no symptoms and optimal function, and 0 indicates severe symptoms and dysfunction [32].

An endurance assessment of the ankle PF muscles was conducted using the heel-rise test. Participants were allowed to touch the wall with their toes for balance. Heel raises were performed at a rate of 30 beats per minute with a metronome [33]. The tested leg remained straight while the other leg was lifted off the floor. Heel raises over 5 cm were counted. The test ended when the participant could no longer perform heel raises due to fatigue, and the total number of raises was recorded [34].

### Surgical technique and postoperative management

All procedures were performed under spinal or general anesthesia with the patient in the supine position. A dorsal longitudinal incision was made over the second tarsometatarsal joint, allowing access to the Lisfranc articulation. Reduction was achieved under fluoroscopic guidance, followed by fixation tailored to the specific injury configuration. Transarticular fixation was performed using fully threaded cannulated screws, typically placed across the intercuneiform joint and between the medial cuneiform and the base of the second metatarsal. Additional fixation of the first tarsometatarsal (TMT) joint was performed in five patients using transarticular screws based on intraoperative assessment of stability. In one patient, arthrodesis of the first TMT joint was performed using a medial plate and screws due to comminution and instability of the joint surface. In this case, an additional medial incision was made to allow for proper plate placement. Postoperatively, all patients were immobilized in a non-weight-bearing short leg cast for the first six weeks. At the sixth week, patients were transitioned to a controlled ankle movement (CAM) boot with toe-touch weight-bearing only, under strict instructions to avoid any significant axial loading. Full weight-bearing was initiated at the twelfth postoperative week, depending on clinical and radiographic signs of healing. No structured physical therapy program was implemented; patients were instructed on gradual weight-bearing and range-of-motion exercises under physician guidance.

### Statistical analysis

The statistical analysis was conducted using the IBM SPSS software (version 25, IBM, Armonk, NY). A priori power analyses indicated that a sample size of ten participants for each group was required, based on the forefoot-hindfoot push-off phase degree with a power of 80% and an alpha level of 0.05, as per van Hoeve et al. [9]. The normality of the parametric data was evaluated using the Shapiro-Wilk test. The data are presented as mean ± standard deviation for parametric variables and as median (minimum-maximum) for nonparametric variables. To compare the two groups, the difference between the two sides of each participant was determined. All subjects in the control group were right-footed dominant. For the control group, the side differences were obtained by subtracting the test results of the dominant side from the test results of the non-dominant side. For the experimental group, the test result of the unaffected side was subtracted from the Lisfranc-injured side [35]. Statistical comparisons were conducted using Student’s t-test for parametric variables and the Mann-Whitney U test for nonparametric variables. Additionally, Cohen’s d effect sizes were calculated for outcomes that demonstrated normal distribution (parametric variables) to complement p-values and assess the magnitude of between-group differences. Effect sizes were not reported for nonparametric variables. The threshold for statistical significance was set at *P* < 0.05.

## Results

### Baseline characteristics and group comparisons

Table 1 presents the descriptive statistics and the statistical comparisons between the groups. Patients in Group I, the majority of whom had left foot injuries, received surgical treatment for Lisfranc injuries. The postoperative follow-up duration for Group I patients ranged from 20 to 42 months. Healthy individuals without a history of orthopedic disorders who were matched for age and sex made up Group II (Table 1).

**Table 1:** Baseline characteristics and statistical comparisons

Variables	Lisfranc Injury (*n* = 12)	Healthy Controls (*n* = 11)	*p*-Value
Age	39.16 ± 13.57	37.00 ± 8.69	0.437
Gender (n, % Male) Side (n, % right) Height Weight	9 (75%) 11 (91,7%) 172.16 ± 12.20 77.16 ± 13.69	8 (%72.7%) 11 (%100%) 175.00 ± 8.16 81.00 ± 15.21	0.903 0.907 0.872 0.848
BMI (kg/m^2^)	26.04 ± 5.91	26.45 ± 5.55	0.870
Myerson classification	Type B1 ◊ 1 patient (8%) Type B2 ◊ 11 patients (92%)		

Continuous variables are presented as mean ± standard deviation (Student t test); categorical variables as n (%) (chi-square test). According to the Myerson classification, 11 patients (91.7%) had B2-type injuries and 1 patient (8.3%) had a B1-type injury

### Changes in foot sensation

In the group I, there was a significant decrease in midfoot protective sole sensation compared to the non-injured side (*p* = 0.031) (Table 2). Compared to the group II, patients with Lisfranc injuries also showed a significant decrease in midfoot protective sole sensation (*p* = 0.019) (Table 3). Although the mean midfoot scores were similar between the injured and uninjured sides, differences in the distribution of scores led to a statistically significant result.

**Table 2:** Within-Group Comparisons For Participants With Lisfranc İnjury And Control Group

Dependent Variables	Lisfranc Injury (*n* = 12)			Healthy Controls (*n* = 11)		
	İnjured Side Uninjured Side *p*			Right Left *p*		
**Foot Sensation (FSS) Tests**						
FSS Hallux (gr)	3.61 (3.61–4.56)	3.61 (3.61–4.31)	0.345	2.83 (2.83–4.31)	3.61( 2.83-0.4.31)	0.715
FSS 1. metatarsal head (gr)	3.61 (2.83–4.31)	3.61 (2.83–4.31)	0.534	3.61 (2.83–4.31)	3.61 (2.83–4.31)	1.
FSS 5. metatarsal head (gr)	4.31( 3.61–4.56)	3.61 (3.61–4.31)	0.274	3.61 (2.83–4.31)	3.61 (2.83–4.31)	0.665
FSS mid foot (gr)	3.61 (3.61–4.31)	3.61 (2.83–4.31)	0.031*	2.83 (2.83–3.61)	2.83 (2.83–3.61)	1.
FSS heel (gr)	4.31 (4.31–4.56)	4.31 (4.31–4.56)	0.356	3.61 (2.83–4.31)	3.61 (2.83–4.31)	1.
**Ankle Proprioception Tests**						
DF AP 7 (^0^)	3.11 (1.56–11.40)	1.78 (0.90–3.83)	0.004*	1.96(0.7–3.60)	1.53 (0.6–5.83)	0.718
PF AP 7 (^0^)	12.48 (5.80–14.80)	2.16 (0.65–9.43)	0.000*	2.79 ± 1.82	3.28 ± 1.25	0.477
PF AP 14 (^0^)	9.81 ± 4.54	2.78 ± 1.65	0.000*	3.56 (1.06–4.03)	2.64 (1.43-5.0)	0.980
PF AP 21 (^0^)	8.2 ± 5.05	4.11 ± 2.96	0.025*	3.06 (1.3–8.06)	2.6 (0.9-10.83)	0.308
**Isokinetic Strength Tests**						
DF PT 30 (Nm)	32.08 ± 9.84	39.3 ± 7.59	0.056	43.72 ± 11.1	39.45 ± 8.61	0.326
PF PT 30 ( Nm)	64.5 ± 19.35	74.75 ± 22.72	0.247	98 ± 25.94	91 ± 13.16	0.464
DF PT 120 (Nm)	18 (8–20)	22 (18–27)	0.004*	24.63 ± 6.69	22.81 ± 3.89	0.448
PF PT 120 ( Nm)	32.8 3 ± 12.72	44.58 ± 12.79	0.034*	51.81 ± 13.45	48.90 ± 9.91	0.571
**Functional Tests**						
WBLT (cm)	7.66 ± 4.00	10 ± 3.07	0.124	12 (10–16)	12 (9–16)	0.893
HRT	14(5–26)	20 (15–30)	0.013*	28.27 ± 3.52	28.36 ± 4.5	0.958
**Balance Tests**						
MSEBT Anterior (%)	66.91 ± 7.68	74.04 ± 10.54	0.072	90.9 ± 13.34	89.27 ± 9.94	0.748
MSEBT Posteromedial (%)	87.5 (22–97)	89 (33–109)	0.086	109.90 ± 18.37	108.72 ± 17.48	0.879
MSEBT Posterolateral (%)	76.12 ± 13.65	83.12 ± 15.08	0.246	96.72 ± 10.97	98.18 ± 10.48	0.754
MSEBT Composite (%)	80 (43–95)	83 (47–94)	0.132	99.09 ± 11.79	98.63 ± 9.74	0.933

Abbreviations: FSS: Foot Sole Sensation, DF: Dorsiflexion, PF: Plantarflexion, AP: Ankle Proprioception, PT: Peak Torque, HRT: Heel-Rise Test, WBLT: Weightbearing Lunge Test, MSEBT: Modified Star Excursion Balance Test

* are the significant differences

Result are presented mean ± standard deviation (Student t test) / med (min-maks) (Mann-Whitney U Test)

**Table 3:** Comparisons between groups for participants with Lisfranc injury and control group

Dependent Variables	Lisfranc Injury (*n* = 12)	Healthy Controls (*n* = 11)	*p*	d
**Foot Sensation (FSS) Tests**				
FSS Hallux (gr)	0 (0-0.7)	0 (-0.78-0)	0.050	-
FSS 1. metatarsal head (gr)	0 (-0.78-1.48)	0 (0–0)	0.639	-
FSS 5. metatarsal head (gr)	0 (-0.7-0.7)	0 (-0.78-0.7)	0.360	-
FSS mid foot (gr)	0 (0-1.48)	0 (0–0)	0.019*	-
FSS heel (gr)	0 (0-0.25)	0 (0–0)	0.166	-
**Ankle Proprioception Tests**				
DF AP 7 (^0^)	1.5 (-0.7-9.84)	0.47 (-4.03–1.47)	0.049*	-
PF AP 7 (^0^)	7.56 ± 4.19	-0.48 ± 1.75	0.000*	2.50
PF AP 14 (^0^)	7.03 ± 4.88	0.02 ± 1.2	0.000*	1.97
PF AP 21 (^0^)	4.08 ± 6.12	0.39 ± 2.37	0.073	-
**Isokinetic Strength Tests**				
DFPT 30 (Nm)	-7.25 ± 6.39	4.27 ± 4.56	0.000*	2.08
PFPT 30 (Nm)	-10.25 ± 20.76	6.54 ± 17.67	0.050	0.87
DFPT 120 (Nm)	-4.5 (-15- 1)	0 (-3-10)	0.001*	-
PFPT 120 (Nm)	-11.75 ± 11.37	2.90 ± 11.97	0.007*	1.25
**Functional Tests**				
WBLT (cm)	-2 (-8-5)	0 (-1-1)	0.001*	-
HRT	-6.58 ± 4.94	-0.09 ± 2.87	0.001*	1.61
**Balance Tests**				
MSEBT Anterior	-7.12 ± 5.75	1.63 ± 5.12	0.001*	1.61
MSEBT Posteromedial	-4 (-16-25)	6 (-19-10)	0.281	-
MSEBT Posterolateral	-7.0 ± 10.50	-1.45 ± 6.72	0.151	0.63
MSEBT Composite	-7 (-9-4)	-1 (-5 -7 )	0.021*	-

Abbreviations: FSS: Foot Sole Sensation, DF: Dorsiflexion, PF: Plantarflexion, AP: Ankle Proprioception, PT: Peak Torque, HRT: Heel-Rise Test WBLT: Weightbearing Lunge Test, MSEBT: Modified Star Excursion Balance Test

* are the significant differences

Efect size are presented as Cohen’s d values (d)

Result are presented mean ± standard deviation (Student t test) / med (min-maks) (Mann-Whitney U Test)

### Impairments in proprioception

In the assessment of the joint position sense between the two ankles in the group I, there was significant impairment in the joint position sense of the injured side at all angles, 7^0^DF, 7^0^PF, 14^0^ PF, and 21^0^ PF. (*p* = 0,004, *p* < 0,001, *p* < 0,001, *p* = 0,025, respectively) (Cohen’s d values were not calculated for within-group comparisons.) (Table 2).

When comparing the joint position sense of the group II with the group I, significant impairment was observed at the 7° DF (*p* = 0.049), 7° PF (*p* < 0.001, Cohen’s d = 2.50), and 14° PF (*p* < 0.001, Cohen’s d = 1.97) angles. No significant difference was detected at the 21° PF angle (*p* = 0.73) (Table 3).

### Muscle strength and endurance

In the group I, With the numbers available, no significant difference could be detected, the two extremities in PF and DF strength at 30 degrees/sec peak torque (*p* = 0,056, *p* = 0,24, respectively) (Table 2) However, there was significantly lower PF and DF endurance at 120 degrees/sec peak torque in the injured foot compared to the opposite extremity (*p* = 0,004, *p* = 0,034, respectively) (Cohen’s d values were not calculated for within-group comparisons.) (Table 2).

When the group I was compared with group II, with the numbers available, no significant difference could be detected in PF strength at 30 degrees/sec peak torque (*p* = 0.05; Cohen’s d = 0.87). However, there was a significant loss in DF strength at 30 degrees/second peak torque and in both PF and DF endurance at 120 degrees/second peak torque in the group I compared to healthy participants (*p* = 0.007, *p* = 0.001, respectively; Cohen’s d = 2.08, 1.25, respectively) (Table 3).

### Dynamic balance deficit

With the numbers available, no significant difference could be detected across all trials of the star excursion balance test scores in the group I (anterior *p* = 0.072, posteromedial *p* = 0.086, posterolateral *p* = 0.246, composite *p* = 0.132) (Table 2). However, when comparing the star excursion balance test scores of the group I with the group II, there was a significant impairment in the balance of patients with Lisfranc injuries in the anterior direction (*p* = 0.001; Cohen’s d = 1.61) and composite balance (*p* = 0.021) (Table 3).

### Reduced ankle mobility

In the group I, with the numbers available, no significant difference could be detected in foot mobility between the injured and uninjured extremities (*p* = 0.124) (Table 2). However, a significant decrease in ankle mobility was observed in the group I compared to the group II (*p* = 0.001; Cohen’s d = 1.61) (Table 3).

### Functional outcomes

The mean AOFAS score for the group I was 75.08 ± 15.47, indicating moderate functional recovery. Regarding the FAOS subscales, the mean scores were 84.58 for Pain, 89.5 for Symptoms, 87.75 for Activities of Daily Living (ADL), 76.58 for Sports and Recreation (Sport/Rec), and 46.66 for Quality of Life (QoL) (Table 4).

**Table 4:** Functional outcomes of patients with Lisfranc injuries

Variables	Lisfranc injury (*n* = 12)
FAOS-Pain	84.58 (range, 46–100)
FAOS- Symptoms	89.5 (range, 56–100)
FAOS- Activities of daily living	87.75 (range, 53–100)
FAOS- Sports/recreation	76.58 (range, 35–100
FAOS- Quality of life	46.66 (range, 6–88)
AOFAS Mid-foot Score	75.08 ± 15.47

Result are presented mean ± standard deviation (Student t test) / med (min-maks) (Mann-Whitney U Test)

## Discussion

This study provides a detailed analysis of foot and ankle function, focusing on the various changes expected to affect these functions following injury compared to healthy controls. To the best of our knowledge, this is the first study to evaluate sole sensation and ankle proprioception in patients with Lisfranc injury.

In the study by Eceviz et al. pedobarographic analysis of patients following Lisfranc injury was performed [7]. It was observed that the maximal force and contact time of the midfoot on the injured side increased compared to the uninjured side. The current study found that protective sole sensation in the midfoot was decreased both on the injured side compared to the uninjured side and when compared to healthy participants. Increased contact time and maximal force may be related to impaired sole sensation in the midfoot of the Lisfranc-injured side. Joint and muscle injuries can cause a loss of proprioception in the affected area, which may lead to functional impairment [17, 18, 36]. In the group I, a decrease in proprioception was observed at all measured angles compared to the group without injury. In addition, a significant impairment in proprioception was found in all angles except 21^0^ PF compared to the healthy participants. These deficits, in our opinion, may stem from the absence of structured rehabilitation following surgery. Mehlhorn et al. highlighted that even in cases where rehabilitation was implemented, residual neuromuscular deficits persisted, correlating with reduced muscle strength and proprioceptive accuracy [8]. This reinforces the necessity of incorporating proprioceptive training into post-operative care plans for Lisfranc injuries.

In patients with Lisfranc injuries, decreased PF and DF endurance were noted on the affected side, whereas DF strength remained statistically unchanged. This finding contrasts with several follow-up studies on foot and ankle injuries, which indicate that PF muscle strength typically declines earlier than DF [37, 38]. For example, Hirschmüller et al. observed a significant loss in PF strength in patients recovering from intra-articular calcaneal fractures, highlighting the susceptibility of PF muscles to early degeneration following injury [37]. Similarly, Stevens et al. reported that prolonged immobilization due to ankle fractures leads to more pronounced strength losses in PF muscles than in DF muscles [38]. However, Mehlhorn et al. found strength deficits in both DF and PF muscles after Lisfranc injuries, with no significant difference between the two groups of muscles [8]. Our results are inconsistent with these findings, as DF strength remained statistically unchanged, while PF and DF endurance showed significant reductions. This discrepancy may be attributed to the target angular velocities in isokinetic testing. The current study evaluated muscle strength at angular velocities of 30°/s, representing maximal voluntary contraction, and endurance at 120°/s, mimicking the higher angular velocities observed in daily activities and gait cycles. Previous studies using different testing protocols may have emphasized muscle performance under varying conditions. For instance, slower velocities often measure peak strength, whereas faster velocities may better capture endurance deficits, particularly in muscles with a more significant functional load during dynamic movements [23, 26]. This highlights the need for standardized testing protocols across studies to ensure comparability and accurate interpretation of findings. When comparing our patient group to healthy controls, both PF and DF strength and endurance were significantly diminished, indicating that Lisfranc injuries can cause persistent impairments despite surgical treatment and restoring daily living activities. These findings suggest that surgical intervention alone may be insufficient to restore muscle function fully, emphasizing the critical role of targeted rehabilitation programs.

Rehabilitation should include specific strategies to enhance PF and DF strength and endurance, particularly given the functional importance of these muscles in activities like walking, running, and climbing stairs. Proprioceptive and balance training may also be incorporated into these programs to address dynamic stability weaknesses, potentially leading to even better recovery outcomes [27, 39]. Similarly, the reduced isotonic endurance in the injured limb, as seen in our heel-rise test results, mirrors findings from studies on patients recovering from Achilles tendon ruptures and ankle fractures, where endurance deficits persisted long after surgical intervention [40–42]. It is essential to remember that the contralateral extremity may also be affected by the injured side’s diminished strength and endurance. According to a cross-effect theory by Mehlhorn et al., compensatory overuse and consequent strength reductions on the unaffected side could result from functional constraints on one side [8]. Bilateral training is crucial in recovery to lessen these consequences and prevent additional difficulties.The long-term disadvantages that patients with Lisfranc injuries experience are further highlighted by dynamic balance abnormalities, especially in the anterior direction. According to Plisky et al., these balance issues, when paired with decreased muscle endurance, greatly raise the likelihood of secondary injuries, such falls or overuse syndromes in the limb that is not injured [27]. When compared to healthy controls, our study’s findings of dynamic balance impairments and endurance deficits indicate that these patients continue to have functional disadvantages even 20 months after surgery.

The AOFAS midfoot scores found in this investigation are consistent with prior findings, demonstrating comparable functional recovery across studies despite methodological variances [9, 43, 44]. However, FAOS ratings were lower than those of previous research, including Fan et al. [44], especially in the quality-of-life area. The lack of a rehabilitation program may be the cause of this discrepancy, which may hinder patients’ capacity to fully recover their capability. The disparity in recovery results implies that variables like the extent of the damage and the absence of post-operative therapy probably have a big impact. The long-term advantages of customized rehabilitation programs to address these deficiencies and improve functional and quality-of-life outcomes should be the main focus of future study. In the study conducted by Zeng et al., significant improvements in AOFAS scores were observed in patients who underwent surgical treatment combined with a rehabilitation program for Lisfranc injuries [45]. Following a minimum of 12 months, patients’ average AOFAS score was 90.45 ± 3.42, indicating the efficacy of treatment regimens backed by rehabilitation. However, in our study, which measured AOFAS scores at an average of 20 (20–42) months after surgery, patients who only received surgical therapy did not show the expected improvement in AOFAS scores. This suggests that more than just surgery could be necessary to achieve full functionality.

This study shows that at an average of 20 months post-surgery, people with Lisfranc injuries still exhibit substantial functional deficits compared to healthy individuals, despite significant advancements in surgical techniques. These results highlight how important rehabilitation is for treating long-lasting deficits in proprioception, muscular endurance, and balance. Targeted activities that increase muscular strength and endurance, improve proprioception, and restore dynamic balance should be a top priority in rehabilitation programs. To avoid compensatory overuse and the ensuing loss of strength in the unaffected limb, bilateral training is also crucial [8]. To learn more about the course of healing, future research should examine the long-term effects of Lisfranc injuries. To further improve patient treatment, prospective studies evaluating how organized rehabilitation programs affect functional and quality-of-life outcomes are crucial.

There are several limitations on this study. The capacity to demonstrate connections between variables is limited by the retrospective design, which may include possible biases such recollection bias. Although the study included a thorough examination of proprioception and balance, a more thorough assessment of postoperative impairments is limited by the absence of functional tests such plantar pressure distribution or gait analysis. The small sample size, mainly resulting from the rarity of partially incongruent Lisfranc injuries treated with ORIF and strict inclusion criteria, may limit the generalizability of the findings. Lastly, there is inconsistency about the durability and therapeutic significance of these impairments over time because long-term functional results and patient satisfaction were not evaluated. In order to better understand recovery trajectories and enhance rehabilitation techniques, future research should incorporate prospective designs and long-term evaluations.

In summary, while Lisfranc injuries significantly impair muscle endurance, particularly in PF and DF groups, the absence of a significant strength deficit in DF muscles suggests a nuanced recovery pattern. Future studies should explore the long-term impact of these deficits and evaluate the efficacy of structured rehabilitation protocols in restoring muscle function and preventing further injuries.

## Conclusion

Despite surgical intervention, individuals with Lisfranc injuries exhibited notable deficits in proprioception, ankle mobility, and muscular strength and endurance, particularly in the DF/PF muscle groups. These deficits led to impairments in dynamic balance, potentially compromising functional recovery and increasing the risk of subsequent injuries. Rehabilitation programs are crucial for addressing these challenges. To maximize recovery outcomes, post-operative treatment should incorporate targeted therapies aimed at improving proprioception, balance, and muscular strength. These findings underscore the importance of individualized and comprehensive rehabilitation plans for enhancing functional outcomes and overall well-being in patients with Lisfranc injuries.

## Data Availability

All data is available from the corresponding author upon reasonable request. Ethical approval: The study was approved by the Clinical Research Ethics Committee of Istanbul University Istanbul Faculty of Medicine (approval number 2271851).
